# Spent Coffee Grounds as Building Material for Non-Load-Bearing Structures

**DOI:** 10.3390/ma15051689

**Published:** 2022-02-24

**Authors:** Tala Moussa, Chadi Maalouf, Christophe Bliard, Boussad Abbes, Céline Badouard, Mohammed Lachi, Silvana do Socorro Veloso Sodré, Lina Bufalino, Fabien Bogard, Fabien Beaumont, Guillaume Polidori

**Affiliations:** 1 Matériaux et Ingénierie Mécanique (MATIM), University of Reims Champagne Ardenne, CEDEX 2, 51687 Reims, France; chadi.maalouf@univ-reims.fr (C.M.); boussad.abbes@univ-reims.fr (B.A.); celine.badouard@univ-reims.fr (C.B.); mohammed.lachi@univ-reims.fr (M.L.); fabien.bogard@univ-reims.fr (F.B.); fabien.beaumont@univ-reims.fr (F.B.); guillaume.polidori@univ-reims.fr (G.P.); 2 Structure Fédérative de Recherche Condorcet FR CNRS 3417, University of Reims Champagne Ardenne, 51100 Reims, France; christophe.bliard@univ-reims.fr; 3 Institut de Chimie Moléculaire de Reims, ICMR-UMR 7312 CNRS, University of Reims Champagne Ardenne, CEDEX 2, 51687 Reims, France; 4 Pôle de Recherche Châlonnais, Université de Reims Champagne-Ardenne, 51000 Châlons-en-Champagne, France; 5 Instituto SocioAmbiental e dos Recursos Hidricos (ISARH), Universidade Federal Rural da Amazônia, 2150-Curio Utinga, Belém 66077, Brazil; silvana.veloso@ufra.edu.br; 6 Instituto de Ciências Agrácias (ICA), Universidade Federal Rural da Amazônia, 2150-Curio Utinga, Belém 66077, Brazil; linabufalino1@gmail.com

**Keywords:** spent coffee grounds, potato starch, bio-based composite, acoustical properties, mechanical properties, hygrothermal properties

## Abstract

The gradual development of government policies for ecological transition in the modern construction sector leads researchers to explore new alternative and low environmental impact materials with a particular focus on bio-sourced materials. In this perspective, the mechanical, thermal insulation, and the sound absorption performances of a spent coffee grounds/potato starch bio-based composite were analyzed for potential application in buildings. Based on thermal conductivity and diffusivity tests, the coffee grounds waste biocomposite was characterized as an insulating material comparable with conventional thermal insulation materials of plant origin. Acoustical tests revealed absorption coefficients in the same range as other conventional materials used in building acoustical comfort. This bio-sourced material presented a sufficient compressive mechanical behavior for non-load-bearing structures and a sufficient mechanical capacity to be shaped into building bricks. Mechanical, thermal, and acoustic performances depend on the moisture environment. The groundwork was laid for an initial reflection on how this composite would behave in two opposite climates: the continental climate of Reims in France and the tropical climate of Belém in Brazil.

## 1. Introduction

Global energy consumption has grown rapidly over the past decade. In 2018, the average growth rate was about 2.3%, nearly twice as high as 2010 [1]. This increase is due to population growth, rapid urbanization, and economic development, leading to high demand for heating and cooling worldwide, accompanied by a 1.7% increase in CO_2_ emissions. Even if, due to the COVID-19 pandemic, primary energy demand dropped by nearly 4% in 2020 and global energy-related CO_2_ emissions decreased by 5.8% [2], a huge increase in energy demand is expected in the forthcoming years, especially in the building sector. 

As a result, successive thermal regulation governmental policies developed since the first oil crisis are pushing towards eco responsibility by considering ecofriendly composite materials in different building applications in line with a sustainable development approach. The role of such composite materials is to reduce the consumption of fossil-based materials, limit greenhouse gas emissions, and create new economic sectors. It is the reason why, in the concept of circular economy, raw or byproducts from renewable resources such as hemp, beet bulb, cork, typha, straw, miscanthus, and flax [3,4,5,6,7,8,9] are studied by transforming low added value co-products into a valuable source of materials in the construction field [10,11,12,13]. According to this rationale, the French environmental regulation RE2020, which comes into force in 2021, should make the use of wood and bio-based materials almost systematic by 2030, including for single-family homes and small apartment buildings, according to the Ministry of Ecological Transition [14]. This process should reduce the carbon footprint of buildings throughout their life cycle, from construction to demolition, while ensuring optimal comfort for occupants.

**Figure 1:**
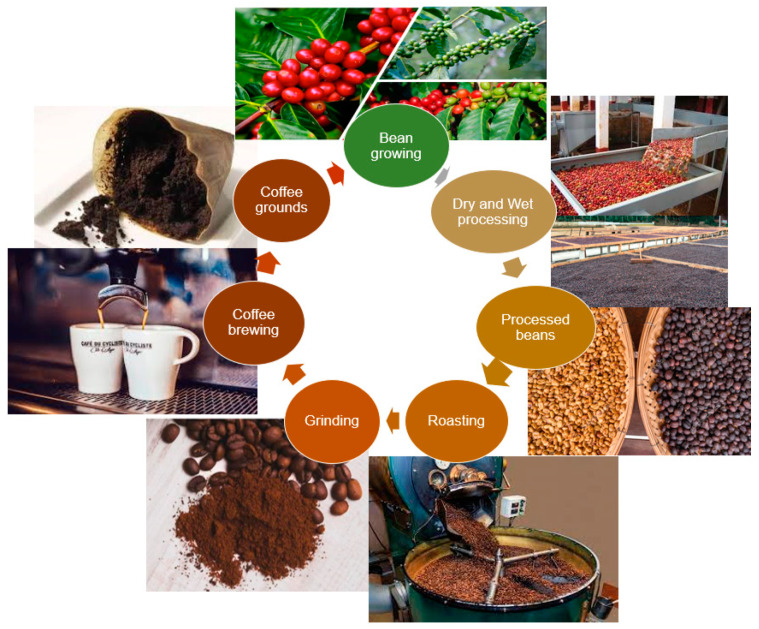
The life cycle of coffee.

Coffee is the second-largest market in the world after oil and a highly traded beverage consumed worldwide. It provides a daily income for 135 million people, including 20 to 25 million small producers in more than 50 developing countries [15]. According to the International Coffee Organization [16], the world coffee consumption corresponded to 166.3 million 60 kg bags in 2020–2021; the first exporting country being Brazil (50.7 million 60 kg-bags) and the first importing country being the European Union (40.2 million 60 kg-bags). The SCA (Specialty Coffee Association) estimates 300 kg of coffee consumption per second. The coffee life cycle from beans to spent grounds is described in Figure 1. Spent coffee grounds (SCG) are the solid-waste products of coffee consumption. Six million tons of coffee grounds are produced per day worldwide [17]. As a result, large quantities of SCG are incinerated, and stored in landfills, potentially releasing harmful substances such as polyphenol and tannin into the environment. Due to their interesting physical and chemical properties [18], there are several possibilities of SCG recovery and reuse, and some authors highlighted their potential in the civil engineering domain [19].

Concerning mechanical and environmental tests, Arulrajah et al. [20] have investigated the application of SCG as a non-structural road fill material. From an environmental perspective, coffee grounds were found to pose no environmental and leaching issues for use as an embankment fill material. From an engineering material perspective, the high organic content, low maximum dry densities, and high optimum moisture content restrict the usage of this material to non-structural fill applications where the material will not have to sustain high traffic loadings. Sena da Fonseca et al. [21] evaluated the potential application of incorporating SCG in clay to manufacture ceramic bricks. Clay pastes containing 5%, 10%, 15%, and 20% coffee grounds were used to mold ceramic samples. The results showed that the inclusion of coffee grounds could increase the apparent porosity and water absorption. Muñoz Velasco et al. [22] and Lachheb et al. [23] studied the improvement of building thermal insulation materials by the addition of SCG. They found a significant decrease in the thermal conductivity with an increase in SCG content. It is reduced by 50% when adding 17% of SCG in fired clay bricks [22] and could be decreased from 0.5 to 0.31 W/(m·K) by adding 6% by weight of coffee grounds in plaster composites. Eliche-Quesada et al. [24] incorporated waste products such as coffee grounds and olive mill residue in clay bricks, and the results seemed to be beneficial. The compressive strength was similar to the value of the reference bricks, and a 19% improvement in thermal conductivity was achieved. Yun et al. [25] investigated the acoustic performances of SCG panels. Their work revealed that the high compressibility and the microscopic layered structure offered a large specific surface area that allowed the absorption of transmitted sound; hence, SCG becomes a viable addition to prefabricated noise barriers [26]. 

Few studies have been conducted to characterize SCG and their feasibility when mixed with a vegetal or mineral binder for construction applications. In this focus, this paper deals with the fabrication of a new starch-bonded spent coffee composite material (SSCC) and its mechanical and physical properties for possible application in the building sector. 

## 2. Materials and Methods

### 2.1. Granulometry of Spent Coffee Grounds

**Figure 2:**
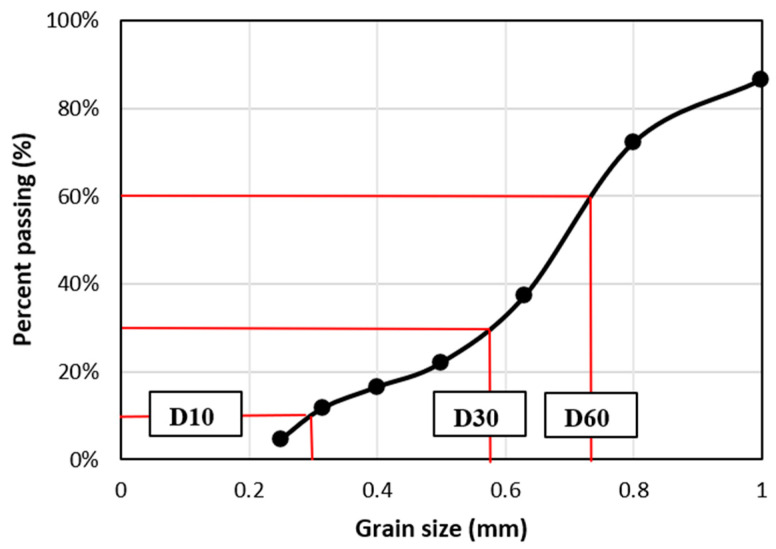
Grading curve of SCG.

The spent coffee grounds (SCG) were collected from various restaurants in Reims (France) and immediately dried for several days in an oven at 70 °C to remove excess moisture. The granulometry was measured on the dried SCG by passing through a series of sieves from 1 to 0.25 mm. Figure 2 shows the weight % passing particle versus the sieve size gradation. Conforming to the geotechnical classification of soils according to the French standard NFP 18-540, the coffee grounds grain-size distribution can be characterized by the uniformity coefficient (Cu), curvature coefficient (Cc), and effective size (D10) from the distribution curve. Cu and Cc can be obtained from the mesh size passing 10% (D10), 30% (D30), and 60% (D60) of the initial SCG weight sample [27], where Dx corresponds to the mesh size of the sieve allowing x% of the weight of the grains to pass through the sieve.

(1) $$\mathrm{Cu}=\frac{D60}{D10}\;\mathrm{and}\;\mathrm{Cc}=\frac{{\left(D30\right)}^{2}}{D10\cdot D60}$$

From Figure 2 it was found that D60 = 0.73 mm, D30 = 0.58 mm, and D10 = 0.29 mm for the coffee grounds samples, leading to Cu = 2.5 and Cc = 1.6 from Equation (1). These values indicate that the SCG samples used in the present study have a uniform and tight grain size with a dominant grain size between 0.3 and 0.8 mm. The rough surface of coffee grounds particles provides good adhesion with the starch binder.

### 2.2. SSCC Preparation

The starch used in this study was from potatoes. It was purchased from Roquette (Lestrem, France). Several compositions were tested by varying the proportion of starch, the binder of the composite materials. Below 40% starch, the SCG composite (SSCC) presented too much friability, preventing their use as insulation material in the construction sector. So, two extreme formulations were prepared to study the composites’ properties, namely, a starch ratio of 0.4 (SSCC-40 friability limit) and 1 (SSCC-100 starch only). The starch/water ratio was set at 0.1 to prepare the paste [28]. Preliminary tests indicated a very brittle constitution of the obtained composites, and glycerol (20% of starch weight) was added to the mix as a starch plasticizer to prevent the sample from being too brittle [29]. In typical SSCC-40 sample preparation, a formulation was prepared by mixing 750 g of SCG with 300 g of starch and 60 g glycerol in 3000 g H_2_O. The mixture was then boiled with a constant and vigorous mechanical stirring until the mix turned into a very viscous paste. At this stage, the hot mix was poured into specific molds to create various sample shapes: 100 × 100 × 100 mm^3^ and 45 × 45 × 45 mm^3^ cubes and 25 mm height × 120 mm or 35 mm diameter circular cylinders. Wood molds were used with a fitted wood piston to compact the mixture inside the mold and contribute to the flatness of the top surface of the specimens. The prepared samples were cooled to 60 °C before being frozen to −30 °C. The frozen samples were pulled out of the molds and freeze dried at 0.6 mbar for several days in a vacuum chamber connected to a Heto PowerDry LL3000 freeze dryer. Final sample dimensions considered a mold shrinkage phenomenon, observed as isotropic with a factor 15/100 for SSCC-100 and 11/100 for SSCC-40. 

### 2.3. Density and Porosity

The bulk density of the composite material is the ratio of the sample mass to the bulk volume which includes the volume of the solids and the voids between the solids. It was obtained with the following formula:

(2) $$\rho_{bulk}=\frac{m}{V}$$

where *m* (kg) is the mass of the aggregates and V (m^3^) is the volume occupied by these aggregates. The different measured bulk densities of the final samples are presented in Table 1. 

The absolute density of the composite material represents the density of the solids without the voids. The absolute density of SSCC-40 and SSCC-100 was determined using the pycnometer method [8]. In the pycnometer, a given mass of the dried sample was placed and then filled with cyclohexane to 2/3 of the total volume of the pycnometer. In this way, the sample was completely covered with cyclohexane, a non-polar solvent that does not affect the composition and mass of the sample. The system was placed under reflux at the boiling temperature of cyclohexane for six cycles of boiling (30 min) and cooling (10 min); during these cycles, air escaped from the sample cavities leaving its pores, and cyclohexane occupied the pore spaces. During the sixth cycle, the system was kept under an argon atmosphere to avoid humidity. After cooling at room temperature, the pycnometer was filled to the top and plugged with the stopper ensuring no bubble was trapped. The system was then weighed with an accuracy of 1 × 10^−3^ g. The absolute density was calculated using the following equation:

(3) $$\rho_{abs}=\rho_{c}\frac{M_{s}}{M_{1}-M_{2}+M_{s}}\;$$

where $\rho_{abs\;}$ is the absolute density, $\rho_{c}$ is the density of the cyclohexane, $M_{s}$ is the mass of the sample, $M_{1}$ is the mass of the pycnometer filled with cyclohexane, and $M_{2}$ is the mass of the pycnometer with cyclohexane and the sample. 

The porosity was calculated as:

(4) $$Porosity=100\;\frac{\rho_{abs}-\rho_{bulk}}{\rho_{bulk}}$$

In the experiments, the porosity was found to be respectively 57.6% for SSCC-40 and 89.3% for SSCC-100.

### 2.4. Mechanical Testing

The Instron 33R 4204 testing machine, equipped with a 50 kN load cell, was used for compression tests. The tests were performed in the same direction as the specimen compaction, according to the ASTM D1621 standard used to determine the compressive properties of rigid cellular materials [30] under indoor conditions of 23 °C room temperature and 50% relative humidity with a rate of 10 mm/min. After isotropic mold shrinkage, starch samples with dimensions 85 × 85 × 85 mm^3^ and SSCC-40 compositions samples with dimensions 40 × 40 × 40 mm^3^ were tested. The apparent Young’s modulus was determined by using Hooke’s law:

(5) σ=E ε

where σ is the engineering stress (MPa), E is Young’s modulus, and ε is the engineering strain (mm/mm). Typically, the apparent Young’s modulus is calculated as the slope of the first linear part of the compression curve σ=f(ε). For each composition, the mean value of three samples was obtained. The apparent Young’s modulus *E* was calculated as the mean value of three samples for each composition.

### 2.5. Thermal Conductivity

Thermal properties were obtained from the commercially produced device ISOMET 2114 (Applied Precision, Ltd., Bratislava, Slovakia) [31], with a measurement accuracy of 5%. This apparatus is a portable device based on the transient hot-wire method used to directly measure heat transfer properties of materials (conductivity, diffusivity, and heat volume capacity). It is equipped with a needle probe implanted in the middle of the sample to be thermally measured. The concept consists in generating a thermal flux by heating an electric resistance inserted in the sample. 

### 2.6. Acoustic Absorption Coefficient

**Figure 3:**
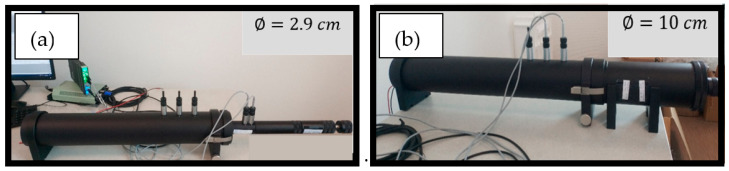
Kundt tube used for high (**a**) and low frequencies (**b**).

A B&K 4206—type of Kundt tube (BKSV, Nærum, Denmark) (tube with two fixed microphones)—was used to measure the sound absorption coefficient α of the composite material. It consists of a cylindrical tube with two quarter-inch BK 4187-type microphones, a power amplifier BK 2735, and an analyzer type 3160-A-042. The Kundt tube measures the sound absorption and the surface impedance according to the standard EN ISO 10534-2. Two different tubes were used to provide a wide frequency range. The device consists of a 10 cm diameter tube for low frequencies (50–1.6 kHz) (Figure 3) and a 2.9 cm diameter tube for high frequencies (500–6.4 kHz). The sound absorption coefficient was measured in the spread frequency range between 50 and 6.4 kHz. Because the acoustic properties depend on the material thickness, especially at frequencies up to 2000 Hz, two thicknesses were tested, namely, 2 cm and 4 cm. Measurements were made on both sides of three circular cylindrical samples and then averaged. The measurement method’s principle lies in studying the transfer function between two signals picked up by two microphones.

### 2.7. Sorption Isotherm

Sorption is the accumulation of water in a hygroscopic material by contact with the environment’s humidity. When the relative humidity of the room air increases at the surface of the material, water is absorbed, increasing the apparent mass. This mass gain, called sorption, is a physical phenomenon that reversibly fixes water molecules. Similarly, a decrease in relative humidity leads to a loss of apparent mass; this is the desorption phenomenon. The sorption isotherm or the hygroscopic curve describes the equilibrium between the water content of the material and the humidity [32]. It can be measured by the continuous or discontinuous method [33]. In this work, the discontinuous method was used. The sorption isotherms were determined according to the European standard EN NF ISO 12571 [34], where the quantity of water adsorbed by the material was measured by increasing the relative humidity. 

Three samples of about 10 g were prepared and dried for one week in an oven at 55 °C. Then, they were introduced in the Binder MK720 climatic chamber at 23 °C, and the relative humidity was fixed at 30%, 50%, 70%, 80%, and 92%. For each humidity level, the samples were weighed daily until the mass increase plateaued. Equilibrium was considered reached once the mass of the sample showed a mass variation of less than 1% on three successive measurements at 24 h intervals. The moisture content by mass was calculated according to the following equation:

(6) $$Moisture\;Content\;\left(\%\right)=100\times \frac{m-m_{0}}{m_{0}}$$

where m and $m_{0}$ are respectively the mass of the equilibrated sample and that of the dry sample.

## 3. Results and Discussions

### 3.1. Mechanical Properties

**Figure 4:**
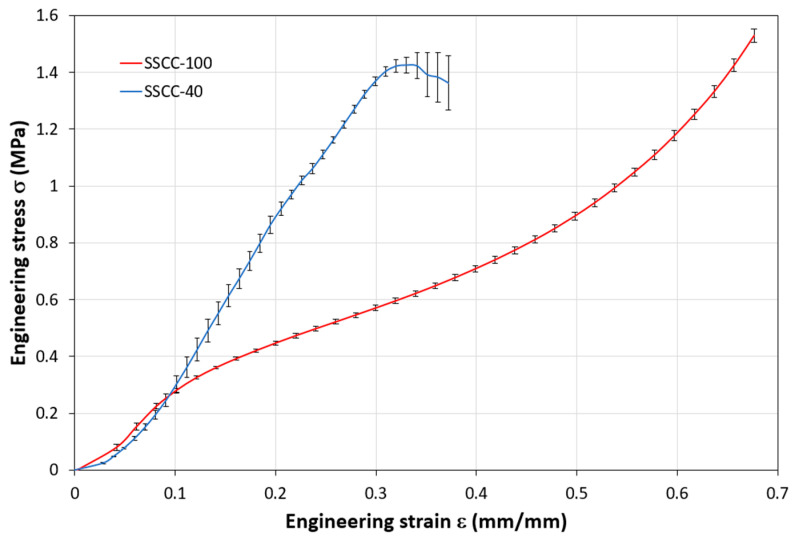
Compression strain–stress curve for SSCC-100 and SSCC-40 samples.

**Figure 5:**
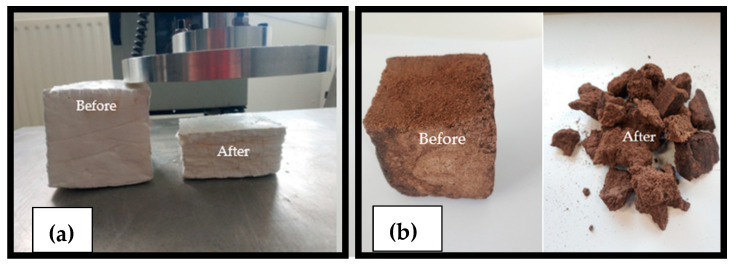
Cube-shaped SSCC-100 (**a**) and SSCC-40 (**b**) features before and after the compressive test.

While knowledge of mechanical properties is not a priori for building materials applied as non-load-bearing structures, it is necessary to ensure that the material will support the weight of the elements above it without collapsing. Results of mechanical compression tests are presented for three samples of each composition: SSCC-100 and SSCC-40. The stress–strain average curve of each composition is presented in Figure 4. The compression process of SSCC-100 exhibits a hyperelastic foam-like behavior divided into three stages: linear elastic stage at low stresses controlled by cell wall bending, followed by a second regime corresponding to the collapse of the cells, truncated by a regime of densification in which the stress rises steeply [35]. For SSCC-40, Figure 4 shows linear elasticity at low stresses, followed by a brittle fracture due to the weakest link mechanism between starch and SCG, as shown in Figure 5.

The compression modulus measures the stiffness of the material or the ability of the material to withstand changes in length when subjected to compressive loads. The higher the compression modulus, the stiffer the material. The different measurements are summarized in Table 1. Results show that Young’s modulus increases with decreasing starch content. For the starch sample, the value was about 3.38 MPa while it increases to 6.09 MPa for the SSCC-40. Young modulus was higher than that of other agromaterials such as beet pulp/starch composites with values ranging from 2.06–3.17 MPa [36], and grape/starch with values varying from 2.52–3.56 MPa depending on the composition [37]. 

**Figure 6:**
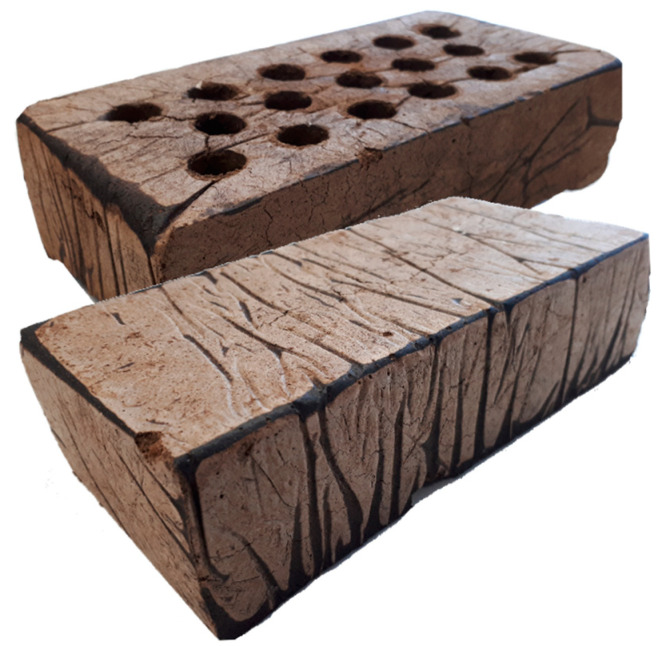
Examples of full and hollowed SSCC-40 bricks (220 × 100 × 60 mm^3^).

Given the rigidity and mechanical properties of this composite material, it becomes possible to create self-supporting walls. As a result, SSCC-40 brick walls can be built to fill the spaces within timber frames, for example, each brick supports the load of those above. In the case of hollowed bricks, Young’s modulus was improved while lowering the weight. Easily masonable solid and hollow brick specimens were created for that purpose from a previously designed molding system [38]. In the case of hollowed bricks, Young’s modulus was improved while lowering the weight. Examples of manufactured full brick and hollowed brick obtained by this process are shown in Figure 6.

### 3.2. Thermal Conductivity 

For insulation materials, thermal conductivity λ is one of the essential properties. The thermal diffusivity *a* is the capacity of a material to transmit heat inside through itself, more or less rapidly. It is customary to consider that excellent thermal insulation materials have a λ less than 0.060 W/(m·K).

These two thermal parameters were measured for three cubic samples (100 × 100 × 100 mm^3^) of each composition, namely, SSCC-100 and SSCC-40. Corresponding average and SD values are given in Table 1. It is noticeable that SSCC-100 has a low thermal conductivity of 0.066 W/(m·K). Adding coffee grounds to starch in a proportion of 60% of the weight increases the thermal conductivity by 33%, reaching a value of 0.093 W/(m·K) for SSCC-40. This value remains nevertheless very acceptable and within the range of thermal conductivities of composite materials such as wood-fiber insulation material with λ = 0.05 W/(m·K), cork with λ = 0.039 W/(m·K), hemp with λ = 0.115 W/(m·K), or sisal fiber with λ = 0.070 W/(m·K) [39]. The rate of transmission of heat flow through the materials studied is characterized by their thermal diffusivity *a*. The lower the thermal diffusivity inside a material, the longer the heat transfer will be and the better the thermal inertia. It was observed that SSCC100 and SSCC40 depicted similar and very low diffusivities of around 0.40 × 10^−6^ m²/s, analogous to materials traditionally used in construction such as rock wool.

In construction, R, the recommended thermal resistance in walls for a low-energy building, should be equal to or greater than 4 m^2^K/W. R is deduced from R = e/λ where e is the thickness (m) of the material. An SSCC-40 composite wall would have to be 37.2 cm thick to meet this recommendation. Decreasing this thickness would demand decreasing the thermal conductivity of the SSCC composite. This requirement could be achieved by adding natural fibers such as flax or nettles fibers in France, and bagasse, açai, or others in Brazil in the initial composite composition.

### 3.3. Acoustic Absorption Coefficient 

**Figure 7:**
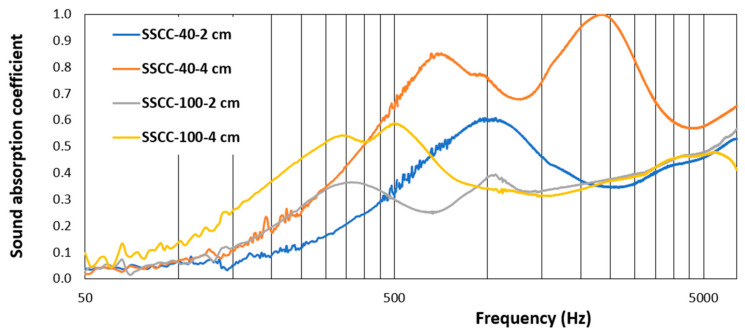
Sound absorption coefficient for starch and SSCC-40 for 2 cm and 4 cm thicknesses.

This new composite, designed for the construction industry, must be a good sound absorber for acoustic comfort and a good thermal insulator. The sound absorption coefficient is used to evaluate the sound absorption efficiency of materials. It is the ratio of absorbed energy to incident energy and is represented by α. If the acoustic energy can be absorbed entirely, then α = 1. The sound absorption evolution is shown in Figure 7 from low to high frequencies for SSCC-100, and SSCC-40 samples as α also depends on the type of binder, porosity structure, and pore distribution in the sample. Figure 7 highlights that as SCG are added to the starch binder, peaks of absorption coefficient shift to the higher frequencies, compared with the reference SSCC-100 with starch only.

Moreover, the SSCC-40 can acoustically reach absorption peaks of 0.6 at 1000 Hz for 2 cm and a double peak of 0.85 at 700 Hz and 1.0 at 2300 Hz for 4 cm. This frequency range corresponds to usual noises in building constructions, meaning that this bio-based material is acoustically efficient for such an application in construction. Compared with other biomaterials, the starch beet composite showed a maximum sound absorption coefficient of 0.72 at 4000 Hz [9] and 0.6 in the medium range. In contrast, hemp starch and cork composites had respectively 0.4 and 0.28 in the medium frequency range [40,41]. Grape stalks composite showed similar behavior with a high value of 0.93 in the range between 1000–4000 Hz [37].

**Figure 8:**
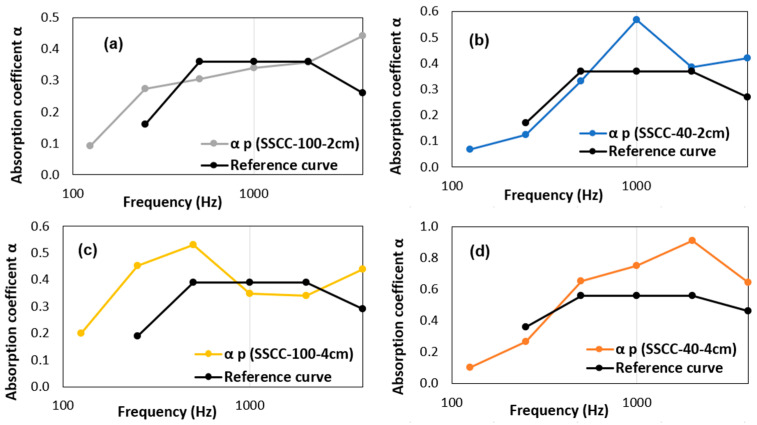
Weighted sound absorption coefficient (α_w_) and practical sound absorption coefficient (αp) for starch (SSCC-100) 2 cm (**a**) and 4 cm thick (**b**); and for SSCC-40 2 cm (**c**) and 4 cm thick (**d**).

Preferred to the noise coefficient reduction (NCR) index considered less accurate, the weighted sound absorption coefficient (α_w_) consists in converting a wide frequency-based range of sound absorption coefficient values into a single number. α_w_ is based on the average of the three one-third octave values over the center band frequency and calculated according to the ISO 11654 standard by comparing the values of the practical sound absorption coefficient (α_p_) based on standard frequencies to a reference curve. The standard’s reference curve must be moved to the curve of (α_p_) in 0.05 increments until the values below the reference curve are less than or equal to 0.1. The value of α_w_ corresponds to the value of the reference curve at 500 Hz. Figure 8 shows the different weighted sound absorption coefficients for the different combinations. For the 2 cm thick SSCC-40 sample, the value was about 0.37, close to that of the starch, and increases to 0.56 for the 4 cm thick sample, while the value for starch remains constant at this same 4 cm thickness. 

The α_w_ coefficient, being higher than 0.5, indicates that the coffee grounds bio-based material presents efficient acoustic properties for the building industry. In Table 2, the comparison of the acoustic properties of SSCC material clearly shows the similarity with the one of rock wool, another material commonly used in construction for acoustic insulation. For a small sample thickness of 2 cm, the SCG composite SSCC-40 was found to have a better-weighted absorption coefficient (α_w_ = 0.37) than the rock wool (α_w_ = 0.25). The opposite phenomenon was observed for a thickness of 4 cm. However, a greater thickness of the SSCC-40 sample, such as that of a manufactured brick, would increase the sound absorption coefficient close to that of the 4 cm thick rock wool [42,43].

**Figure 9:**
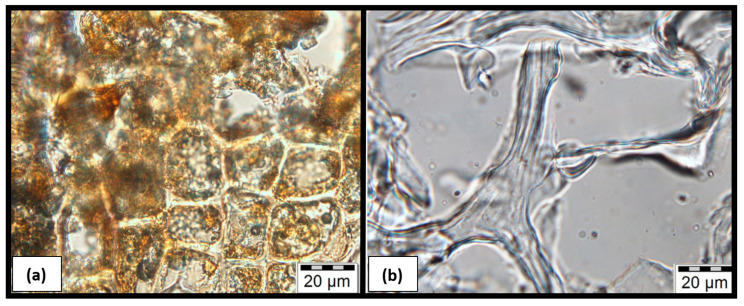
Microscopic photographs of microtome slices of (**a**) SSCC-40 showing dark coffee particles embedded in clear starch gel. (**b**) SSCC-100 displays clear starch gel filaments and large voids (vacuoles).

Microscopic observations by microtome show for SSCC-100, Figure 9b, that filaments of starch aerogel create many vacuoles (thus voids), as suggested by the low bulk density of the material (Table 1). For the SSCC-40, Figure 9a, we observe that these vacuoles are filled with SCG particles; the starch gel is wrapped around the particles, thus resulting in a closed structure. This closed structure explains the interesting acoustic properties of the SSCC-40 composite (Figure 7). The weak characteristics of the SSCC-100 (Figure 4) are explained by the important proportion of these vacuoles and thus of voids in the material.

### 3.4. Sorption Isotherm at T = 23 °C

**Figure 10:**
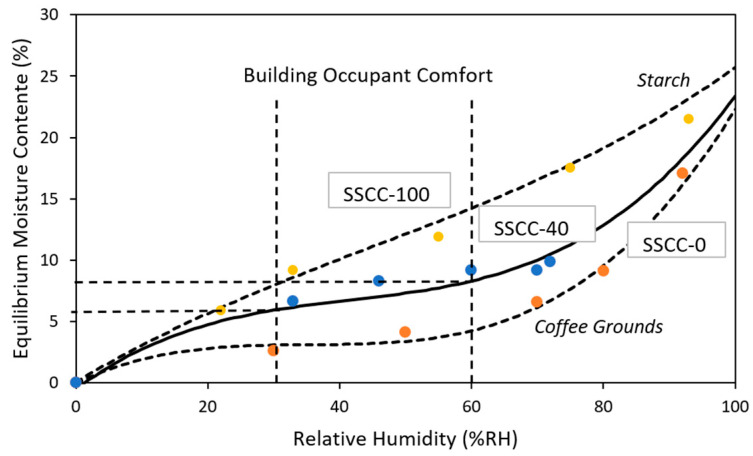
Sorption isotherms of SSCC-100, SSCC-40, and SCG (SSCC-0) at 23 °C after 27 days (%RH).

Prior knowledge of mechanical, thermal, and acoustic properties is necessary to design and develop bio-sourced composite materials in the building sector. However, knowledge alone is not sufficient for a practical application in the construction industry as these materials are moisture sensitive due to their natural hygroscopicity. When bio-based materials are exposed to water vapor of a definite partial pressure, sorption of the water by the composite will occur. Moisture is one of the main drawbacks in building disorders inducing microorganism growth, discomfort, and also degradation in thermal stability and material performance. The consequences of uncontrolled moisture in an ecofriendly material from biowaste management such as SSCC-40 can be contrary to the initial motivations of sustainable development, resulting in excess energy consumption. Sorption isotherms of SSCC-100, SCG, and SSCC-40 were analyzed at 23 °C to understand how moisture accumulates in the material when placed in an equilibrium moisture atmosphere. The results are presented in Figure 10. Fitted curves for SSCC-0 and SSCC-100, deduced from experiments, constitute the upper and lower limit thresholds in the elaboration of the SSCC composite. In this preliminary study, experiments were conducted for a temperature of 23 °C.

Unlike the SSCC-100, which can be approximated by a quasi-linear behavior, moisture sorption isotherm for SCG (SSCC-0) and SSCC-40 exhibits strong non-linear and sigmoidal-shaped behaviors with an exponential type of growth beyond 65% RH. The curve typically corresponds to a type II classification [44] which considers the existence of water multilayers at the internal material surface. Adding coffee grounds to starch at 60% in weight drastically decreases the moisture content. After 27 days, the equilibrium moisture content reaches 17% for each sample for an ambient environment of 92% RH, close to that of the SSCC-100. It was clearly seen that in the range of relative humidity between 30 and 60%, which corresponds to the range targeted for indoor building comfort in both residential and tertiary sectors [45], the equilibrium moisture content for SSCC-40 varies between 6 and 8%. Conventionally, 12% is the maximum moisture content threshold according to the international reference code for composite materials to avoid damaging fungus growth [46].

The results found herein encourage the development of this kind of ecofriendly material for building insulation. Even if this study deals with fully bio-based materials, the determination of the chemical properties of this new material would also provide valuable information in the context of combining biomaterials with cement-based materials. Future studies will focus on this.

## 4. Reflection Tracks and Perspectives

It seems very difficult to dissociate the interior and exterior environments of a building linked to the climate depending on whether it is dry or humid, cold, or hot. Recent experiments and results on thermal-mechanical and acoustic characteristics of SSCC-40 samples are valid to 23 °C ambient temperature and 50% relative humidity only. A fundamental criterion for developing plant-based composites is the climate-dependent dynamics of their intern moisture transfer.

To lay the groundwork and ask questions for future studies on the behavior of this composite in different moisture environments, we will consider two highly humid climates, respectively, cold–temperate and hot: the continental climate of the city of Reims in Northern France and the tropical climate of the city of Belém in Amazonian Brazil.

**Figure 11:**
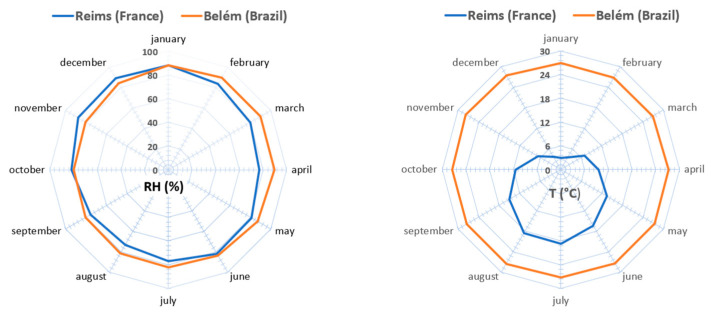
Monthly average relative humidity (**left**) and temperature (**right**) for the Reims (France) and Belém (Brazil) cities.

Climatic characteristics given by both the French Meteo-France agency and the Brazilian Instituto Nacional de Meteorologia are given in Figure 11. If the representation does not show a significant difference concerning the relative humidity, the temperature, on the other hand, differs strongly. In detail, averaged over two decades between 1991 and 2010, the annual relative humidity for Reims was 81.4% (oceanic climate, latitude 49°15′ N) and 84.9% for Belem (tropical climate, latitude 1°27′ S) with slight monthly variation between these two cities. The annual average temperature was respectively 10.6 °C cold for Reims and 27.3 °C hot for Belém with very distinct seasons (cold and snowy winter and warm to hot summer) for Reims, typical of a humid continental climate. 

Inevitably, one can think that an entirely bio-sourced material cannot have identical performances in such different climates. Further studies will have to consider the differences in average temperature between Belém and Reims and their influence on hygrothermal properties. Equilibrium moisture content evolution will have to be established under these particular temperatures, in addition to the setting up of accelerated aging tests in climatic enclosures.

Moreover, if coffee grounds are a waste product found in all four corners of the globe, starch as a plant binder depends on the geographical location of the cultivation and exploitation areas. To leave a smaller carbon footprint, a reflection will have to be carried out on the choice of the starch source with respect to local economic short circuits for the manufacture and transportation of composite bricks: for example, potatoes starch for France and cassava starch for the state of Para in Brazil. Will these different starches induce the same performances at the same dosages? Should the dosages be adapted? The addition of local natural fibers can also be an attractive technological solution to increase the thermal resistance of non-load-bearing walls made of composite coffee grounds while reducing their thickness: flax or nettles for France, and açai, or bagasse for the state of Para in Brazil. All these tracks of reflection will be the subject of future studies.

## 5. Conclusions

This study deals with a practical approach for the management of spent coffee grounds, upcycled for reuse in the building industry as an insulation material. This new biocomposite, associated with a plant-based binder from potato starch, is designed to be a sustainable alternative to the mineral or petroleum-based compounds used for this purpose, which depletes resources. The results reported herein encourage the development of this kind of ecofriendly building material due to its good thermal and acoustic properties. Nevertheless, additional analyses must be carried out on the hydric behavior of this 100% bio-sourced material under different hot and cold humidity environments, such as those encountered in the North French humid continental climate and the Brazilian Amazonian tropical climate.

## Figures and Tables

**Table 1:** Intrinsic thermomechanical properties of starch and SSCC-40% samples.

Samples/Composition	Bulk Density *ρ* (kg/m^3^)	Young’s Modulus *E* (MPa)	Conductivity λ (W/(m·K))	Diffusivity *a* (m^2^/s^−1^)
SSCC-100	161 ± 23	3.38 ± 0.50	0.066 ± 0.004	0.379 ± 0.097 × 10^−6^
SSCC-40	588 ± 12	6.10 ± 0.49	0.093 ± 0.006	0.438 ± 0.054 × 10^−6^

**Table 2:** Comparison between α_w_ values for SSCC-40 and rock wool.

Materials	Ref	Thickness (cm)	α_w_
SSCC-40	-	4	0.56
SSCC-40	-	2	0.37
Rock wool	[42]	4	0.80
Rock wool	[43]	2	0.25

## Data Availability

The study did not report any data.
